# Supplementary material for: HIVprotI: an integrated web based platform for prediction and design of HIV proteins inhibitors
Source: J Cheminform. 2018 Mar 9;10:12. doi: 10.1186/s13321-018-0266-y (PMC5845081; doi:10.1186/s13321-018-0266-y)
Supplement: Supplementary file 2 — Additional file 2. Source code of HIVProtI web server. [file 13321_2018_266_MOESM2_ESM.zip › HIVprotI_Source-code/hivprotI/weka-3-6-6/doc/weka/attributeSelection/SubsetEvaluator.html]

SubsetEvaluator


---


|  |  |  |
| --- | --- | --- |
| **Overview** \| **Package** \| **Class** \| **Tree** \| **Deprecated** \| **Index** \| **Help** \| **Weka's home** | |  |
| **PREV CLASS**   **NEXT CLASS** | **FRAMES**    **NO FRAMES**     **All Classes** |  |
| SUMMARY: NESTED \| FIELD \| CONSTR \| METHOD | DETAIL: FIELD \| CONSTR \| METHOD |  |


---


## weka.attributeSelection Interface SubsetEvaluator

**All Known Implementing Classes:**: CfsSubsetEval, ClassifierSubsetEval, ConsistencySubsetEval, CostSensitiveSubsetEval, FilteredSubsetEval, HoldOutSubsetEvaluator, UnsupervisedSubsetEvaluator, WrapperSubsetEval

---

``` public interface SubsetEvaluator ```

Interface for attribute subset evaluators.

**Version:**
:   $Revision: 1.11 $

**Author:**
:   Mark Hall (mhall@cs.waikato.ac.nz)

---

| **Method Summary** | |
| --- | --- |
| `double` | `evaluateSubset(java.util.BitSet subset)`             evaluates a subset of attributes |

**Method Detail**

### evaluateSubset

```
double evaluateSubset(java.util.BitSet subset)
                      throws java.lang.Exception
```

:   evaluates a subset of attributes

    :   **Parameters:**: `subset` - a bitset representing the attribute subset to be evaluated **Returns:**: the "merit" of the subset **Throws:**: `java.lang.Exception` - if the subset could not be evaluated


---


|  |  |  |
| --- | --- | --- |
| **Overview** \| **Package** \| **Class** \| **Tree** \| **Deprecated** \| **Index** \| **Help** \| **Weka's home** | |  |
| **PREV CLASS**   **NEXT CLASS** | **FRAMES**    **NO FRAMES**     **All Classes** |  |
| SUMMARY: NESTED \| FIELD \| CONSTR \| METHOD | DETAIL: FIELD \| CONSTR \| METHOD |  |


---
